# Pharmacotherapies for fatigue in chronic liver disease (CLD): a systematic review and meta-analysis (protocol)

**DOI:** 10.1186/s13643-018-0688-7

**Published:** 2018-02-14

**Authors:** Andem Effiong, Prerna Kumari

**Affiliations:** 1 Georgetown University, 3700 O St NW, Washington, DC 20057 USA; 2 Manipal College of Pharmaceutical Sciences, Manipal, Karnataka India

**Keywords:** Fatigue, Liver disease

## Abstract

**Background:**

This is the protocol for a systematic review (and meta-analysis) of an intervention. The primary objective of this systematic review will be to assess the benefits and harms of pharmacological therapies (pharmacotherapies) for the management of fatigue in adults with CLD of any etiology. The effects of pharmacological therapies on fatigue in CLD will be compared against those of placebo, no intervention, or non-pharmacological interventions. Specifically, this review will examine whether pharmacological therapies improve CLD-associated fatigue, and if they do, what key elements are associated with their effectiveness. The results of this systematic review will assist clinicians, policy-makers, researchers, and people with CLD in decision-making on how best to manage fatigue and its associated symptoms.

**Methods:**

MEDLINE, SCOPUS, EMBASE, EU Clinical Trials Register, WHO International Clinical Trials Registry Platform, CENTRAL (The Cochrane Library), ClinicalTrials.gov, reference lists of articles and conference proceedings will be searched for relevant studies. No language or date restrictions will be applied. Eligible studies will include adults with CLD of any etiology. Included studies will be randomized controlled trials. From included studies, data on participant characteristics, study design, setting, research ethics compliance, and intervention outcomes will be extracted. Risk of bias in included studies will be assessed using the Cochrane Risk of Bias Tool. A random-effects meta-analysis will be conducted. If substantial or considerable levels of heterogeneity are detected, analysis will be limited to a narrative synthesis.

**Discussion:**

This systematic review will examine the effectiveness of pharmacological therapies on fatigue reduction in people with CLD. Such therapies may be more effective than non-pharmacological interventions in treating fatigue symptoms in CLD. Evidence derived from the findings of this study will guide future practice, policy, and research.

**Systematic review registration:**

PROSPERO, CRD42017076957

**Electronic supplementary material:**

The online version of this article (10.1186/s13643-018-0688-7) contains supplementary material, which is available to authorized users.

## Background

For the purposes of this systematic review, we define chronic liver disease (CLD) as progressive destruction of normal liver functions, commonly associated with fibrotic regeneration of the liver tissue [1]. Diseases which comprise CLD include compensated cirrhosis of any etiology, chronic hepatitis, alcoholic liver disease (ALD), non-alcoholic steatohepatitis (NASH), cholestatic liver disease, and metabolic liver disease. Major underlying causes of CLD vary depending on geographical location. In low- and middle-income countries, etiologies for CLD are predominantly viral, while in western countries and some regions of the Indian subcontinent, alcohol is frequently implicated [2, 3]. In the USA, non-alcoholic fatty liver disease (NAFLD), a form of NASH, and defined as abnormal accumulation (> 5%) of hepatic triglyceride in the absence of excess alcohol consumption, is the most common form of CLD [4]. Because CLD encompasses a broad spectrum of hepatic pathology, different diagnostic tools for screening exist. Diagnostic tools may be invasive (e.g., liver biopsy) or non-invasive (e.g., biochemical markers and imaging techniques) [4, 5]. This review will include studies in which CLD was detected and confirmed with at least one of the following diagnostic tools: laboratory examination, liver biopsy, or radiologic imaging.

Worldwide, CLD is a major contributor of morbidity and mortality and therefore constitutes a significant public health burden [6, 7]. Clinical signs of CLD include jaundice, ascites, gynecomastia, encephalopathy, and spider angioma; however, these signs are not necessarily specific for CLD, and their presence in people with risk factors or other indicators of liver disease may require management strategies encompassing a well-structured liver disease workup to determine their etiology [8].

Fatigue may be acute (self-limiting) or chronic (debilitating and of greater than 6 months duration [9]. Fatigue is a multidimensional symptom often experienced as loss of energy and resulting in disparities between intention and ability. Abnormalities of circadian rhythm, e.g., increased daytime somnolence, have been linked to the pathogenesis of fatigue [10, 11]. Increased central serotoninergic tone is also believed to be a precursor of fatigue in chronic illness. Among patients with CLD, even though fatigue is the most prevalent symptom, its pathogenesis is not well understood. Moreover, the overlap of fatigue in CLD with other neuropsychiatric conditions, such as depression and anxiety, makes objective interpretation and treatment extremely difficult [10, 11]. In CLD, fatigue is thought to originate from the central nervous system (CNS) and is often marked by a reduction in quality of life, partly attributed to challenges in completing daily life activities [11]. CLD patients with decompensated cirrhosis or liver failure may also suffer from peripheral fatigue (characterized by muscle wasting and weakness) as a result of neuromuscular dysfunction. It is worth noting that both peripheral and central fatigue can occur simultaneously [12].

Few pharmacological or drug interventions currently exist primarily for the treatment of symptoms associated with fatigue in people with CLD [13]. Some non-pharmacological interventions, e.g., psychosocial interventions, physical activity, and lifestyle modifications have been found to be effective in reducing fatigue or promoting improved liver function in liver disease; however, their results have been mixed. Although fatigue in CLD is pervasive, there is no established therapy for its management [14]. Pharmacological interventions targeting fatigue reduction in people living with CLD are classified as either specific or non-specific therapies [14]. Accordingly, therapeutic effects of both specific and non-specific interventions will be examined.

Due to the complexities involved in objectively measuring fatigue, assessments of fatigue in people with CLD are usually done with questionnaires (e.g., Short Form-36 (SF-36), Fatigue Severity Scale (FSS), or Fatigue Impact Scale (FIS)) which permit objective quantification or scoring based on an individual’s answers to a particular set of questions regarding fatigue [15, 16]. Alterations in fatigue scores are therefore used to ascertain whether therapeutic interventions facilitate improvement or worsening of feelings or symptoms of fatigue [15, 16].

People with CLD who suffer from frequent fatigue often have numerous functional impairments and lower quality of life [17, 18]. Nonetheless, their experiences and symptoms are often underreported and undertreated [17, 18]. The lack of clarity and dearth of evidence on the effects of pharmacological therapies for the management of fatigue in CLD makes it imperative to conduct a systematic review which further investigates the benefits and harms of these therapies, especially among those who suffer from severe fatigue [19, 20].

## Methods/design

This systematic review protocol was developed in accordance with the Preferred Reporting Items of Systematic Reviews and Meta-Analysis for Protocols (PRISMA-P) 2015 [21]. The completed PRISMA-P checklist is included as an additional file (see Additional file 1). The PRISMA extension for Abstracts (PRISMA-A) was used as a checklist to develop the protocol abstract [22]. This 12-item checklist will also be used as a framework to develop the abstract of the systematic review. In the event of any amendments to this protocol, the date of each amendment and its justification will be updated in the registration record and completed systematic review. The systematic review will adhere to the reporting guidelines of the Preferred Reporting Items for Systematic Reviews and Meta-Analyses (PRISMA) statement [23]. Its methodological quality will be appraised and reported using A Measurement Tool to Assess Systematic Reviews (AMSTAR) 2 [24].

### Objectives

The primary objective of this systematic review will be to assess the benefits and harms of pharmacological therapies (pharmacotherapies) for the management of fatigue in adults with CLD of any etiology. Specifically, this review will examine whether pharmacological therapies improve CLD-associated fatigue, and if they do, what key elements are associated with their effectiveness.

### Review question

Are pharmacological interventions alone or in combination with other pharmacological interventions more effective than placebo, no intervention, or non-pharmacological interventions in the management of fatigue in people with CLD?

### Eligibility criteria

#### Study design

Studies selected for inclusion in this systematic review will be limited to randomized controlled trials. Individual studies must have assessed fatigue as a primary or secondary outcome measure, and not solely as an adverse effect, with results reported independently for CLD. Quasi-randomized controlled trials will be excluded.

#### Participants/population

Eligible populations will include adults with all types of CLD, regardless of severity or time since diagnosis of illness.

#### Interventions

This review will include pharmacological interventions used in the management of fatigue in CLD. Interventions will be included irrespective of delivery setting or person delivering the intervention. Pharmacological interventions will be compared either against each other or placebo (or no intervention) or non-pharmacological interventions.

#### Comparator

- Placebo (or no intervention)
- Other pharmacological interventions
- Non-pharmacological interventions

#### Outcomes

Outcome measurements will be assessed at baseline and post-treatment at different lengths of follow-up. Fatigue will encompass malaise, lethargy, exhaustion, and decreased vitality [10].

- ○ Primary outcomes
-  ● FSS score (lower limit = 1; upper limit = 7; final score = mean value of the 9 items; with lower scores being favorable, i.e., FSS score ≥ 4 being indicative of fatigue) [20].
- ○ Secondary outcomes
-  ● Fatigue scores (other, validated)
-  ● Quality of life
-  ● Adverse events (including all causes of mortality)

### Search strategy

An extensive search of the literature will be conducted in the following databases:

MEDLINE, SCOPUS, EMBASE, Clinicaltrials.gov, EU Clinical Trials Register, WHO International Clinical Trials Registry Platform, and CENTRAL (The Cochrane Library). There will be no date or language limits. Reference lists of eligible studies and conference proceedings will also be searched. The provisional search strategies for MEDLINE and EMBASE are included as an additional file (see Additional file 2).

### Study selection and screening

Titles and abstracts of all potentially eligible studies will be retrieved and screened by both AE and PK. And then, they will both independently screen full texts of studies (that were selected during the initial title and abstract screening) for inclusion in the systematic review.

Any initial disagreements on study selection will be resolved through discussion. In the event that AE and PK fail to agree on study eligibility, a non-author colleague designated a priori as an arbiter will be invited to make a final decision.

### Data extraction and management

AE and PK will independently appraise and extract data using a standardized data abstraction form. The data extraction process for each included study will be guided by AMSTAR 2 recommendations [24] and will include the following information:

- Design, sponsor, conflict of interest statement, aims and objectives, risk of bias, demographic characteristics, and outcomes assessed;
- Number of participants randomized for each study arm, type of placebo, non-pharmacological intervention or pharmacological intervention used in comparator arm, type of pharmacological therapy used in experimental arm, what amounts to no intervention when applicable;
- Doses administered, clinical outcomes (e.g., FSS scores, quality of life, adverse events), and surrogate outcomes (e.g., non-adherence and missing data, i.e., attrition or loss to follow-up).

To minimize the likelihood of selective outcome reporting, all time-points measured for each outcome within each study will be reported [25]. Study investigators will be contacted via email for missing data or confirmation of available data.

### Methodological quality

AMSTAR 2 will be used to assess the methodological quality of the systematic review.

AMSTAR 2 is a validated 16-item instrument that is used to critically appraise systematic reviews of randomized controlled trials [24].

### Risk of bias assessment

The Cochrane Risk of Bias Tool which covers six domains, namely, selection bias (random sequence generation and allocation concealment), performance bias (blinding of participants and personnel), detection bias (blinding of outcome assessment), attrition bias (incomplete outcome data), reporting bias (selective reporting), and other bias (other potential sources of bias) will be used to assess risk of bias for each included trial [26]. The sequence generation process described by the investigators will be assessed to determine whether it was truly random.

For each domain, a judgment of high risk of bias, low risk of bias, or unclear risk of bias will be assigned [26]. Risk of bias will be assessed regardless of any expected variability in study results or validity.

### Quality of the evidence

The quality of evidence obtained will be assessed using the Grading of Recommendations Assessment, Development and Evaluation (GRADE) approach [27]. AE and PK will individually assess the quality of the evidence. Disagreements will be resolved through arbitration by a non-author colleague.

### Data synthesis and analysis

Data will be analyzed using the Cochrane Review Manager (RevMan) 5.3 [28]. We will use a random-effects model for meta-analysis if moderate heterogeneity is detected [29]. Where substantial or considerable levels of heterogeneity are detected, we will limit our analysis to a narrative synthesis [29].

### Measuring treatment effect

Risk ratio or relative risk (RR) will be used as summary statistics for dichotomous outcomes. For continuous data, we will use the mean difference (MD) or standardized mean difference (SMD) as summary statistics calculated as single assessments or change from baseline measures of individual data [30]. If possible, we will summarize time-to-event data by survival analysis 152 and present the intervention effect as a hazard ratio (HR) [25]. Or, we may analyze time-to-event data as dichotomous data if the status for all patients within a study is presented at a fixed time point, and a HR analysis is not feasible [30]. We will present all measures of effect with 95% confidence intervals (CI) [30].

### Units of analysis issues

We will take into account the level at which each randomization occurred. If appropriate, we will consider whether each participant in a trial was subject to more than one intervention, or whether there were different observations for the same outcome, by evenly distributing the comparator and experimental group participants evenly between both groups. Available data from all randomized controlled trial phases will be assessed for suitability for inclusion in the analysis.

### Missing data

Should we encounter missing data, we will attempt to contact study investigators or sponsors to obtain numerical outcome data or validate important study attributes. Where possible, we will conduct analyses based on the intention-to-treat (ITT) principle [30]. If there is loss to follow-up participants, missing outcomes will be excluded from the analysis.

### Assessment of heterogeneity

Heterogeneity across all the studies will be explored by using a combination of these measures:

I. Examination of forest plots and their associated outputs, e.g., Cochran’s Q
II. Determination of *I*^2^ statistic, i.e., variation in effect size due to heterogeneity (with 30–59%, 60–89%, and 90–100% representing moderate, substantial, and considerable levels of heterogeneity)
III. Determination of *T*^2^ and tau (i.e., estimate of between-study variance or measure of the dispersion of true effect sizes between studies based on the magnitude of the effect size) for determining the prediction interval.

### Subgroup analysis

If appropriate, we will further investigate sources of heterogeneity through analyses by the following subgroups:

I. Age
II. Sex/gender
III. Race/ethnicity
IV. Education
V. Type of CLD

Potential confounders of subgroup effects that we may consider include the following:

- Age
- Comorbidities (e.g., depression, anxiety, post viral infection, unspecified somatoform disorder, chronic fatigue syndrome, thyroid disorder, and anemia)
- Intervention features: intensity, content, delivery
- Length of study

An in-depth analysis of each subgroup will be done by stratification of the effect modifying variable [31]. We will assume that risks are unevenly distributed. To overcome the effects of confounders, we will first adjust for confounders in our statistical analyses within relevant subgroups to quantify their effect size followed by an assessment within subgroups that are stratified by the effect modifying variable [31–33]. Where stratification becomes impractical, we will conduct a multivariate regression [32, 33].

To determine whether perceived differences between subgroups are clinically relevant, we will calculate effect sizes (Cohen’s *d*) and correlations for the main variables, and make comparisons between initial and final values derived from the original sample and subgroups. Determination of the robustness of observed effects will be guided partly by the degree of consistency of our primary outcome (fatigue) analysis over different subgroups. Lastly, we will report on whether each subgroup variable assessed was measured at baseline or after randomization because measurements after randomization may be less credible.

### Sensitivity analysis

If appropriate, we will conduct a sensitivity analysis to test the robustness of our findings. We will evaluate the effects of methodological quality on the pooled estimate by repeating the analysis after excluding trials that were deemed high or unclear risk within at least two domains, namely, performance and detection bias [25].

### Assessment of publication bias

Where possible, reporting and publication bias will be assessed by way of funnel plots created to identify asymmetry [30]. The choice of test for funnel plot asymmetry will be dependent of the degree of heterogeneity observed [30].

## Discussion

This systematic review will examine and synthesize the evidence of the benefits and harms of pharmacological interventions in the management of fatigue in people with CLD of any etiology. Currently, few high quality studies exist that have evaluated the use of pharmacotherapies in the treatment of fatigue in people with CLD.

Such therapies may be more effective than non-pharmacological interventions in treating fatigue symptoms in CLD. Evidence derived from the findings of this study will guide future practice, policy, and research. Study findings will also be disseminated to target audiences in different settings and published in a peer reviewed journal.

## Additional files

Additional file 1: PRISMA-P CHECKLIST. (DOCX 28 kb)

Additional file 2: Search strategy <MEDLINE & EMBASE>. (DOCX 11 kb)

## Abbreviations

ALD: Alcoholic liver disease
AMSTAR: A Measurement Tool for the Assessment of Multiple Systematic Reviews
CI: Confidence interval
CLD: Chronic liver disease
EU: European Union
FIS: Fatigue Impact Scale
FSS: Fatigue Severity Scale
GRADE: Grading of Recommendations Assessment, Development and Evaluation
HR: Hazard ratio
MD: Mean difference
NAFLD: Non-alcoholic fatty liver disease
NASH: Non-alcoholic steatohepatitis
PBC: Primary biliary cholangitis
PRISMA: Preferred Reporting Items for Systematic Reviews and Meta-Analyses
PRISMA-P: Preferred Reporting Items for Systematic Reviews and Meta-Analysis Protocols
RevMan: Review Manager
RR: Risk ratio or relative risk
SMD: Standardized mean difference
WHO: World Health Organization
