# Supplementary material for: Pharmacotherapies for fatigue in chronic liver disease (CLD): a systematic review and meta-analysis (protocol)
Source: Syst Rev. 2018 Feb 14;7:28. doi: 10.1186/s13643-018-0688-7 (PMC5813416; doi:10.1186/s13643-018-0688-7)
Supplement: Supplementary file 2 — Search strategy . (DOCX 11 kb) [file 13643_2018_688_MOESM2_ESM.docx]

**Search Strategy**

Definitions of the various search terms in MeSH (medical subject headings = indexing terms):

Drug Therapy:  <https://www.ncbi.nlm.nih.gov/mesh/68004358>

drug therapy [subheading]:  <https://www.ncbi.nlm.nih.gov/mesh/81000188>

Fatigue:  <https://www.ncbi.nlm.nih.gov/mesh/68005221>

Liver Diseases:  <https://www.ncbi.nlm.nih.gov/mesh/68008107>

Randomized Controlled Trial [publication type]: <https://www.ncbi.nlm.nih.gov/mesh/68016449>

When searching for up-to-date information, we will include keywords in the title and/or abstract because of the indexing delay.  The search limit for keywords in the title and/or abstract is [tiab].  Also, if a searcher wants the term itself and does not want to include the terms under it, then we will use the limit:  [mh:noexp].

***Provisional search strategies***

MEDLINE (I)

(((drug therapy [mh] OR drug therapy [sh]) AND (liver diseases [mh] AND fatigue [mh:noexp])) OR (chronic [tiab] AND "liver disease*"[tiab] AND fatigue [tiab] AND drug* [tiab]) AND randomized controlled trial [pt])

EMBASE (II)

(drug therapy and liver diseases and fatigue and randomized controlled trial)

We will also use the indexing term "Randomized Clinical Trials as Topic":

<https://www.ncbi.nlm.nih.gov/mesh/68016032>
